# Supplementary material for: Preserving the Josephson Coupling of Twisted Cuprate Junctions via Tailored Silicon Nitride Circuits Boards
Source: Small. 2025 Nov 11;21(50):e06520. doi: 10.1002/smll.202506520 (PMC12710119; doi:10.1002/smll.202506520)
Supplement: Supplementary file 1 — Supporting Information [file SMLL-21-e06520-s001.pdf]

NANO · MICRO
small

Supporting Information

for *Small*, DOI 10.1002/smll.202506520

Preserving the Josephson Coupling of Twisted Cuprate Junctions via Tailored Silicon Nitride Circuits Boards

*Tommaso Confalone, Flavia Lo Sardo, Domenico Montemurro, Davide Massarotti, Valerii M. Vinokur, Genda Gu, Francesco Tafuri, Kornelius Nielsch, Golam Haider and Nicola Poccia**

Supplementary Information: Preserving the Josephson Coupling of Twisted Cuprate Junctions via Tailored Silicon Nitride Circuits Boards

Tommaso Confalone,[1,2] Flavia Lo Sardo,[1,3] Domenico Montemurro,[4] Davide Massarotti,[5] Valerii M. Vinokur,[6] Genda Gu,[7] Francesco Tafuri,[4] Kornelius Nielsch,[1,2,3] Golam Haider,[1] and Nicola Poccia[1,4,*]

[1] *Leibniz Institute for Solid State and Materials Research Dresden (IFW Dresden), 01069 Dresden, Germany*
[2] *Institute of Applied Physics, Technische Universität Dresden, 01062 Dresden, Germany*
[3] *Institute of Materials Science, Technische Universität Dresden, 01062 Dresden, Germany*
[4] *Department of Physics, University of Naples Federico II, 80125 Naples, Italy*
[5] *Department of Electrical Engineering and Information Technology, University of Naples Federico II, I-80125 Naples, Italy*
[6] *Terra Quantum AG, 9000 St. Gallen, Switzerland*
[7] *Condensed Matter Physics and Materials Science Department, Brookhaven National Laboratory, Upton NY 11973, USA*

[*] nicola.poccia@unina.it

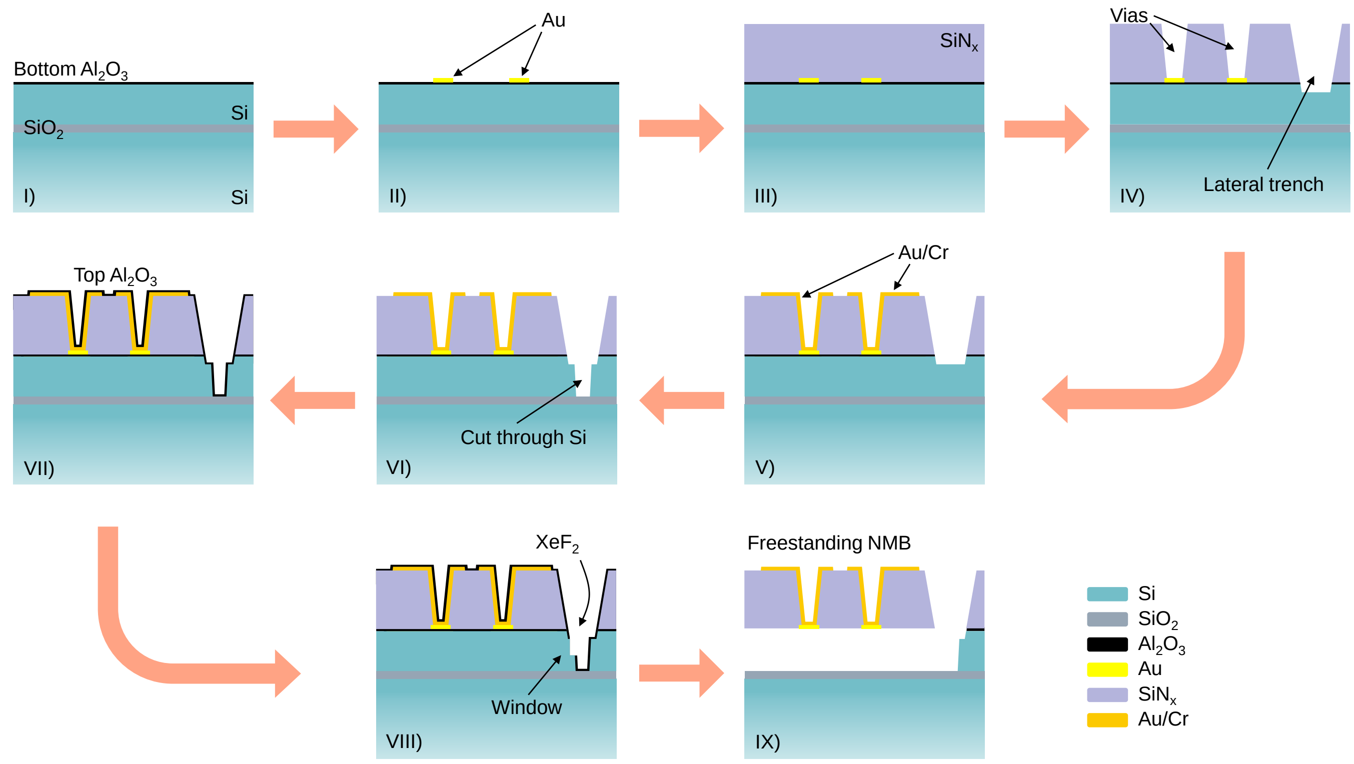

FIG. S1. Schematic cross-section of the steps for the fabrication of the nanomembranes (NMBs). I) Deposition of the bottom Al_2O_3 layer on the Si/SiO_2/Si substrate. II) Realization of the Au bottom contacts. III) Deposition of the SiN_x layer for the body of the membrane. IV) Etching in the SiN_x of the vias for exposing the underlying bottom contacts and at the same time of the trenches that determine the perimeter of the NMBs. V) Sputtering of the Au/Cr stacks for realizing the upper contacts. VI) Etching of the Si to expose the buried SiO_2 layer. VII) Deposition of the Top Al_2O_3 layer to cover completely the NMBs. VIII) Opening windows in the Al_2O_3 layer for the etching of the Si sacrificial layer through XeF_2 vapor and make the membrane freestanding. IX) Removal of all protective Al_2O_3 layers and drying.

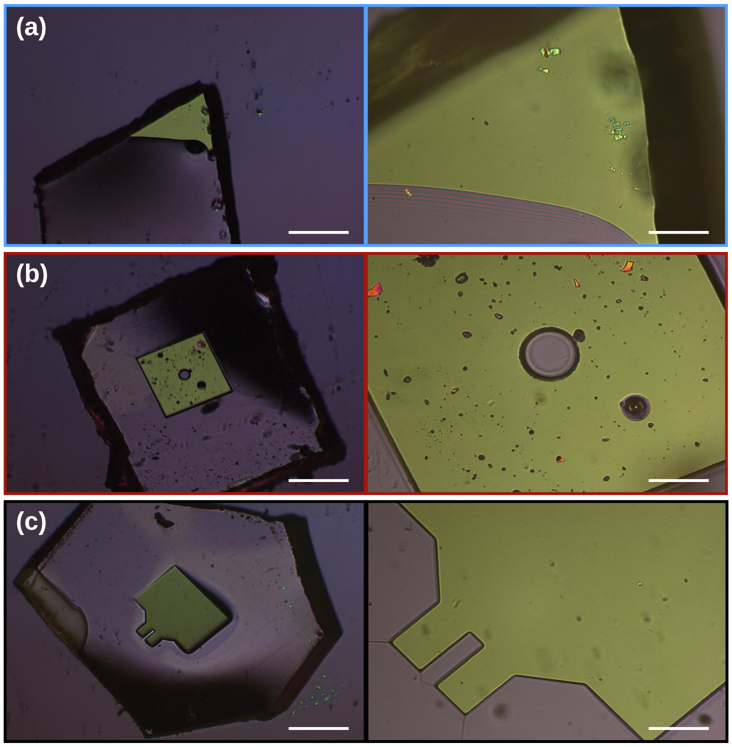

FIG. S2. (a)–(c) Optical microscopy images of the three shaped PDMS structures (covered, with hole, and double cantilever) used to transfer the NMBs, with close-up views of the touching regions, highlighted in moss green. Scale bars represent 500 μm for the full images and 80 μm for the zoomed-in areas.

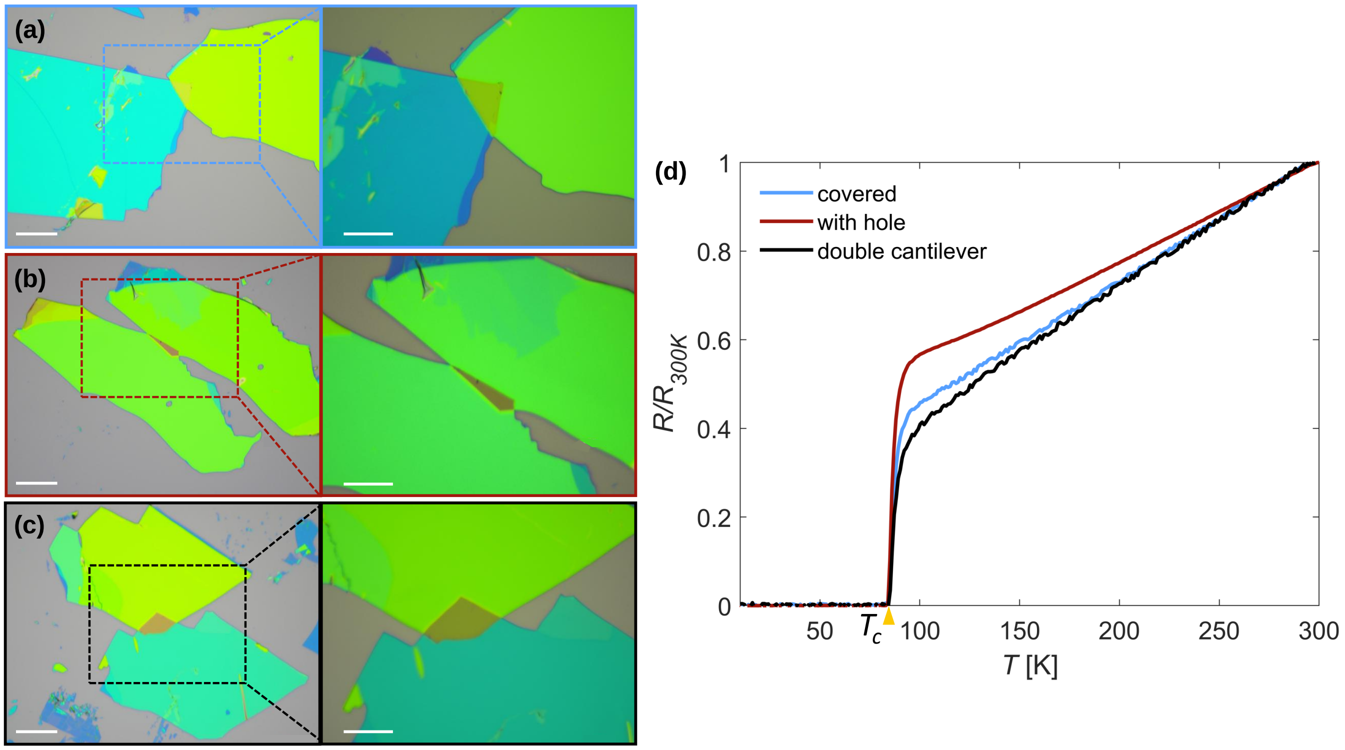

FIG. S3. (a)–(c) Optical microscopy images of the three compared (covered, with hole, double cantilever) untwisted BSCCO devices, with close-up views of the junction regions, prior the landing of the nanomembranes (NMBs). Scale bars represent 50 μm for the full images and 20 μm for the zoomed-in areas. (d) Temperature-dependent electrical resistance normalized at 300 K obtained for the compared untwisted BSCCO junctions. All three samples exhibit a critical temperature of approximately $T_c \approx 84$ K (yellow triangle).

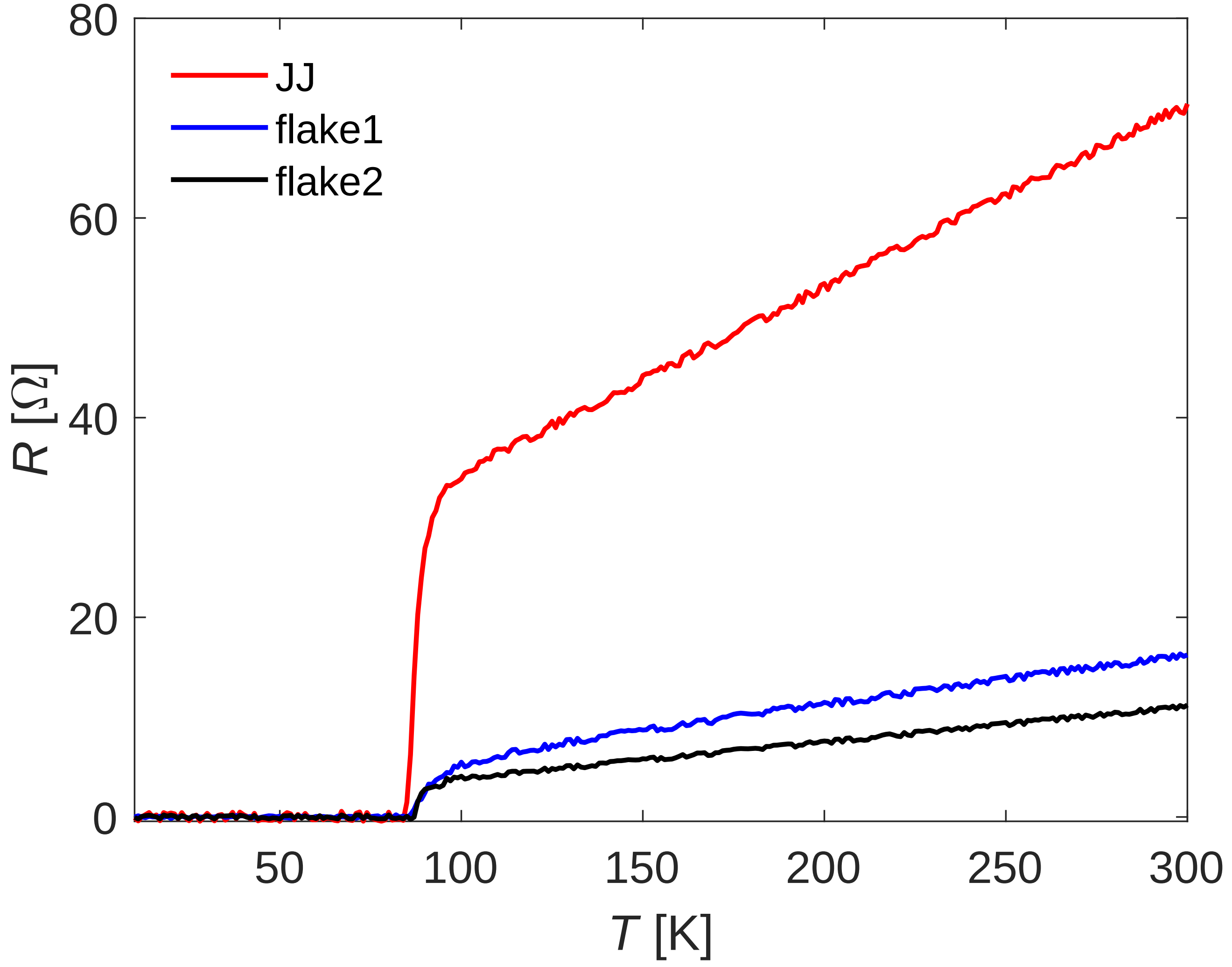

FIG. S4. Temperature dependent resistance across the twisted junction $\theta = 20.5^{\circ}$ (red) and the two flakes (black and blue) composing the JJ.
